# Characterization of *Plasmodium* Lactate Dehydrogenase and Histidine-Rich Protein 2 Clearance Patterns via Rapid On-Bead Detection from a Single Dried Blood Spot

**DOI:** 10.4269/ajtmh.17-0996

**Published:** 2018-03-19

**Authors:** Christine F. Markwalter, Lauren E. Gibson, Lwiindi Mudenda, Danielle W. Kimmel, Saidon Mbambara, Philip E. Thuma, David W. Wright

**Affiliations:** 1 Department of Chemistry, Vanderbilt University, Nashville, Tennessee;; 2 Department of Chemistry and Biochemistry, Elizabethtown College, Elizabethtown, Pennsylvania;; 3 Rusangu University, Monze, Zambia;; 4 Macha Research Trust, Choma, Zambia

## Abstract

A rapid, on-bead enzyme-linked immunosorbent assay for *Plasmodium* lactate dehydrogenase (*p*LDH) and *Plasmodium falciparum* histidine-rich protein 2 (HRP2) was adapted for use with dried blood spot (DBS) samples. This assay detected both biomarkers from a single DBS sample with only 45 minutes of total incubation time and detection limits of 600 ± 500 pM (*p*LDH) and 69 ± 30 pM (HRP2), corresponding to 150 and 24 parasites/μL, respectively. This sensitive and reproducible on-bead detection method was used to quantify *p*LDH and HRP2 in patient DBS samples from rural Zambia collected at multiple time points after treatment. Biomarker clearance patterns relative to parasite clearance were determined; *p*LDH clearance followed closely with parasite clearance, whereas most patients maintained detectable levels of HRP2 for 35–52 days after treatment. Furthermore, weak-to-moderate correlations between biomarker concentration and parasite densities were found for both biomarkers. This work demonstrates the utility of the developed assay for epidemiological study and surveillance of malaria.

## INTRODUCTION

Quantitative laboratory measurement of malarial protein biomarkers helps define disease prevalence, distribution, and infection intensities. In the context of malaria elimination, sensitive detection methods are useful for determining response to interventions on a population level, ensuring that low-density infections are identified, and informing the development of improved point-of-care diagnostics. However, the logistics and biohazard risk of venous whole blood sample collection, preservation, and transportation from the field to the laboratory often pose challenges to large studies.

Many of these challenges are mitigated by the use of dried blood spot (DBS) cards for sample collection and preservation. In this sampling technique, which does not require specialized skills or equipment, microliter volumes of whole blood collected from a finger prick are spotted onto filter paper cards and allowed to dry at room temperature. These DBS samples are then easily stored or shipped, pose little biohazard risk, and result in improved biomarker stability compared with liquid samples.^1,2^ The DBS cards are often cost-effective compared with venous whole blood sample tubes and require no instrumentation to carry out the minimally invasive collection procedure.^3^

Because of these advantages, DBS sample cards have been used extensively in surveillance and epidemiological studies of malaria. For example, extraction and detection/sequencing of nucleic acid material from DBS have allowed for not only malaria detection in symptomatic and asymptomatic patients^4–6^ but also speciation,^7^ determination of parasite diversity,^8^ identification of drug-resistant strains,^9,10^ and evaluation of rapid diagnostic tests on a population level.^11–13^ Dried blood spots have also been used for the detection of antimalarial antibodies for serology-based epidemiological studies.^14^

Quantitation of malarial protein biomarkers from DBS samples, although less common than nucleic acid detection, is also relevant in the context of malaria elimination. Recently, two studies have measured *Plasmodium falciparum* histidine-rich protein 2 (HRP2), the primary protein biomarker used to diagnose malaria, in DBS patient samples. Rogier et al.^15^ used their DBS detection method to evaluate the accuracy of HRP2-based rapid diagnostic tests, and Gibson et al.^16^ demonstrated the persistence of HRP2 in patient DBS samples after treatment compared with microscopy. Although both of these studies demonstrated sensitive HRP2 quantitation from DBS, there are several disadvantages of using HRP2 alone as a diagnostic marker for malaria. First, HRP2 persists in host circulation for several weeks after parasite clearance, potentially resulting in false-positive results and unnecessary prescription of antimalarials.^17^ Second, HRP2 is only expressed by one of the five species of malaria known to infect humans. Third, infections with *P. falciparum* histidine-rich protein 2 (*pfhrp2*) gene deletions are increasing in prevalence and result in false-negative results on HRP2-only tests, posing a major challenge for case management.^18^

To address these disadvantages, we previously developed a highly sensitive, magnetic bead–based assay that simultaneously captured and sequentially detected *Plasmodium* lactate dehydrogenase (*p*LDH) and HRP2 from a single whole blood sample.^19^ Because *p*LDH is a metabolic enzyme required for parasite survival, it is present during infections from any of the five species of malaria known to infect humans.^20,21^ In addition, *p*LDH has been shown to clear within days after treatment.^22^ As such, this simultaneous capture and sequential detection (SCSD) assay not only distinguishes between *falciparum* and non-*falciparum* infections but also differentiates between active and resolved *falciparum* infections. In addition, the lower limit of detection of the SCSD assay, 2.0 parasites/μL for both *p*LDH and HRP2, was an order of magnitude improved over commercially available enzyme-linked immunosorbent assay (ELISA) kits and would allow for detection of individuals with asymptomatic or submicroscopic malaria infections.^19^

In this work, we adapt the previously developed SCSD assay for *p*LDH and HRP2 to detect these biomarkers from DBS and apply it to patient samples from rural Zambia. In particular, the clearance patterns of both biomarkers relative to parasite clearance are investigated. The high sensitivity of this assay is ideal for DBS sample analysis because these samples consist of just a few microliters of whole blood diluted into extraction buffer. In addition, the total protocol requires only 45 minutes of total incubation time for quantitation of both biomarkers, increasing the throughput and information yield per sample.

## MATERIALS AND METHODS

### Materials.

Human whole blood (K3 EDTA) was purchased from BioreclamationIVT (Hicksville, NY) (catalog no. HMWBEDTA3). Recombinant HRP2 protein was generously provided by PATH (Seattle, WA). Recombinant *P. falciparum* lactate dehydrogenase (rc*Pf*LDH) was purchased from CTK Biotech (San Diego, CA) (Catalog no. A3005). *Plasmodium falciparum* D6 strain was cultured in the laboratory (stock concentration 18,450 parasites/μL or 43,600 parasites/μL). Anti-HRP2 antibodies were purchased from Abcam (Cambridge, United Kingdom) (ab9203, ab9206, and ab30384). Anti-*p*LDH antibodies were purchased from Vista Diagnostics (Kirkland, WA) (19g7 and 1201). Vista 1201 was conjugated to alkaline phosphatase (1201:AP) using Abcam ab102850 and to horseradish peroxidase (1201:HRPx) using Thermo no. 31489. BluePhos^®^ microwell phosphatase substrate was purchased from KPL (Gaithersburg, MD) (No. 50-88-02), and TMB One was purchased from Promega (Madison, WI) (G7431). Dynabeads^®^ MyOne^™^ streptavidin T1 beads were purchased from Life Technologies (Carlsbad, CA) (no. 65601). Immulon 2HB ELISA plates (14-245-61) were purchased from Fisher Scientific (Pittsburgh, PA). 903 Protein saver cards were purchased from GE Healthcare Life Sciences (Marlborough, MA) (10534612). Six-millimeter biopunches were acquired from Ted Pella Inc. (Redding, CA) (catalog no. 15111-60). All other reagents were purchased from either Fisher Scientific or Sigma Aldrich (St. Louis, MO). Dried blood spot extraction was performed with a Fisher Scientific analog vortex mixer (02-215-365). Absorbance measurements were collected on a Biotek Synergy H4 microplate reader (Vanderbilt University) or Biotek ELx808 microplate reader (Macha Research Trust).

### Standardization of D6 *P. falciparum* culture.

Two stocks of in-house D6 *P. falciparum* culture (18,450 parasites/μL and 43,600 parasites/μL) were used in this study. The *p*LDH and HRP2 concentrations in the 18,450 parasite/μL stock were previously reported as 1.3 and 1.7 pM per parasite/μL, respectively.^16,19^ In addition, HRP2 in the 43,600 parasites/μL stock was previously determined to be 2.2 pM per parasite/μL.^23^ The *p*LDH concentration of in-house D6 *P. falciparum* culture (stock 43,600 parasites/μL) was determined to be 4.4 pM per parasite/μL using a standard well-plate ELISA, *N* = 6 (Supplemental Text 1).

### Dried blood spot preparation and extraction.

Dried blood spot extraction was adapted from a previously reported method.^16^ Dried blood spot was prepared by depositing 10 μL of parasitized whole blood onto Whatman 903 protein saver cards. The spots were allowed to air-dry for a minimum of 4 hours, removed using a 6-mm biopsy punch, and placed in 2-mL microcentrifuge tubes (one spot per tube). Next, 200 μL of phosphate-buffered saline (PBS) with 0.1% Tween-20 (PBST) was added to each tube. The tubes were vortexed at 3,200 rpm for 10 minutes and then placed in a mini-centrifuge for 30–60 seconds. The supernatant was removed and saved for analysis.

### Bead preparation.

Anti-*p*LDH and anti-HRP2 beads were prepared as reported previously.^19,24^ Briefly, anti-*p*LDH (Vista 19g7) or anti-HRP2 (Abcam ab9203) was biotinylated with EZ-Link NHS–PEG4–Biotin, No-Weigh Format (Thermo no. 21329). Unreacted NHS–PEG4–Biotin was removed using 7K MWCO Zebra spin desalting columns (Thermo no. 89882). Next, 5 mg of Dynabeads MyOne streptavidin T1 was washed three times with PBS and incubated with 500 μL of 0.4 mg/mL biotinylated antibody in PBS for 30 minutes. The beads were then washed before incubating in excess D-biotin in PBS for 30 minutes. Finally, the beads were washed and resuspended in 500 μL of PBS with 0.01% Tween-20. Stock solutions of antibody-functionalized beads were transported to Zambia in ambient conditions and stored at 4°C on arrival.

### Simultaneous capture and sequential detection ELISA with DBS extracts.

**Figure 1.**
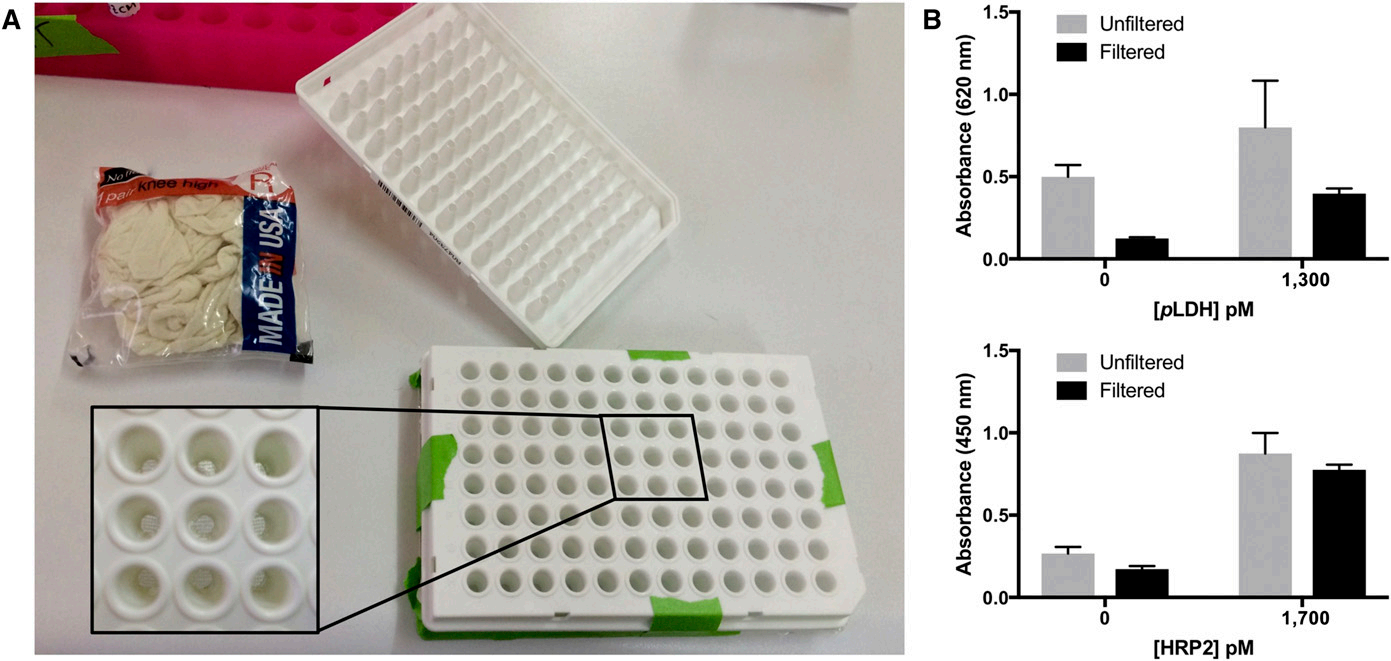
(**A**) Affordable 96-well plate filter for use in low-resource settings. (**B**) Filtering improved the performance of both the *Plasmodium* lactate dehydrogenase (*p*LDH) and *Plasmodium falciparum* histidine-rich protein 2 (HRP2) portions of the dried blood spot simultaneous capture and sequential detection ELISA. This figure appears in color at www.ajtmh.org.

The SCSD ELISA on DBS extracts was adapted from the previously reported method.^19^ To avoid bead aggregation, a low-resource–filtering method was devised for removing small fibers and paper pieces from DBS extracts (Figure 1). Nylon fabric (Walmart, Bentonville, AK, No Nonsense Knee Highs) was cut to the appropriate size and taped onto a Fisherbrand flat-bottom PS 96-well plate (no. 12565501). A polymerase chain reaction (PCR) plate with the bottoms of the wells removed was then taped on top of the flat-bottom plate such that the nylon fabric was taut across the bottom of each well, forming a nylon filter between the wells of the flat-bottom and PCR plates. Next, 100 μL of DBS extract was pipetted through the nylon fabric filter into the flat-bottom plate. The nylon fabric and PCR plate were removed, and 100 μL of 10% nonfat dried milk in PBST was added to each well, followed by 4 μL of HAMA blocking reagent (Fitzgerald 85R-1001), 10 μL of 19g7-conjugated magnetic beads, 5 μL of ab9203-conjugated magnetic beads, 1.57 μL of 1201:AP (1.27 mg/mL), and 2 μL of ab30384 (0.1 mg/mL). The plate was protected from light and incubated on an orbital shaker for 15 minutes. A MagWell^™^ magnetic separator was used to remove the supernatant and wash the beads twice, first with 200 μL of PBST followed by 100 μL of PBST. On the second wash, the beads were moved to new wells, and 100 μL of BluePhos microwell phosphatase substrate was added to each well. The plate was protected from light and incubated for 15 minutes. The absorbance of the supernatant was measured at 620 or 630 nm (*p*LDH detection). Next, the beads were washed three times (100 μL PBST) and moved to new wells on the last wash before being resuspended in 100 μL TMB One solution and incubated for 5 minutes. The supernatant was then removed and the reaction was quenched with 100 μL of 2 M H_2_SO_4_. Absorbance was measured at 450 nm for detection of HRP2.

### Stability study.

Dried blood spots were prepared and stored in Ziploc bags containing the desiccant at room temperature (up to 8 days) and −20°C (up to 188 days). At varying time points, DBSs were removed from storage and analyzed using the SCSD ELISA for *p*LDH and HRP2.

### Study setting.

Patient DBS samples were collected from the Nchelenge district of Zambia as part of a separate study on parasite clearance rates in children less than 5 years of age presenting with uncomplicated malaria at a local clinic. These de-identified samples were made available to the authors for assessment of *p*LDH and HRP2 clearance patterns relative to parasite clearance rates using the DBS SCSD ELISA.

### Patient recruitment and ethics.

Children at the clinic who tested positive for malaria (SD Bioline Pf) were recruited for this study only if a parent or guardian provided written informed consent. The samples were collected under institutional review board approval TDRC/C4/09/2014 and after approval was granted by the Zambian National Health Research Authority (MH/101/17/6).

### Patient samples.

Finger-prick blood samples were collected on protein saver 903 cards. At the time of collection, parasitemia was determined by thick smear microscopy; parasites were counted per 200 white blood cells (WBC) and parasite levels were determined using an estimate of 8,000 WBC/μL. Samples were collected between December 2014 and August 2015, stored at −20°C, and analyzed by SCSD ELISA in July 2016. Patients were enrolled in the study and received treatment with artemether–lumefantrine (Coartem^®^) after malaria diagnosis by SD Bioline Pf RDT and confirmation of infection by thick smear. Samples (DBS and thick smears) were then collected at 15 time points after treatment: 0, 6, 12, 18, 24, 30, 36, 42, and 48 hours as well as 3, 7, 14, 21, 28, and 35 days. Samples for all time points for 15 patients were analyzed in this study.

### Patient DBS sample SCSD ELISA.

All patient samples were coded, and the assays were carried out blinded to microscopy results. Patient DBS samples were extracted and analyzed via the SCSD ELISA as described previously with the following exceptions: 1) the standard curve (0–400 parasites/μL from 18,450 parasites/μL stock: 0–520 pM *p*LDH, 0–680 pM HRP2) consisted of 1:19 (v:v) parasitized whole blood diluted in PBST, mimicking the matrix of DBS extract, and 2) if the signal for either *p*LDH or HRP2 was above the linear range of the assay, the DBS extract was reanalyzed at the appropriate dilution.

### Data analysis.

Biomarker concentrations in DBS extracts were interpolated from best fits of linear standard curves. All error bars shown are the standard error of measurement. Limits of detection were calculated as the biomarker concentration at *s*_blank_ + 3SD_blank_. Intra-assay variation (%CV) was determined as the average relative standard deviation of triplicate measurements on a single plate. Inter-assay variation (%CV) was determined by finding the standard deviation of all measurements at a given concentration on different days and dividing by the average absorbance measurement at that concentration. For analysis of clearance rates across all patients, biomarker concentrations were normalized to their highest value across all time points for each patient.

## RESULTS AND DISCUSSION

### Dried blood spot SCSD ELISA optimization.

The protocol for the DBS sample SCSD ELISA was optimized systematically. Optimum conditions for HRP2 recovery from DBS were previously reported.^16^ To determine whether this method achieved sufficient elution of *p*LDH, the recoveries of both biomarkers were compared across multiple extraction times in PBST. The *p*LDH extraction efficiencies were not significantly different from those of HRP2 across all DBS extraction incubation times. In addition, increasing time did not result in significant differences in recoveries for either biomarkers (Supplemental Figure 1).

Once the DBS samples were extracted, the eluents were filtered to reduce nonspecific signal due to bead aggregation around small fibers and pieces of paper. To accomplish this, we developed an affordable, homemade filtering device that could be used in low-resource settings (Figure 1A). The filter consisted of a 96-well PCR plate with the tips of the tubes cut off. Cheap, commercially available nylon fabric covered the open bottoms of the PCR plate, which nested directly into a flat-bottomed 96-well plate. The nylon fabric was discarded after all samples and standards were filtered into the flat-bottomed plate, and the PCR plate was washed in 10% bleach, followed by three washes with deionized water, and reused with fresh nylon fabric for filtering. The total cost of the filtering device was $0.10/sample, but recycling the PCR plate decreased filtering costs to as low as $0.012/sample. As shown in Figure 1B, filtering DBS extracts through this device reduced nonspecific background signal by 4-fold for *p*LDH and 1.5 times for HRP2, increasing the signal-to-noise ratio from 1.6 to 3.2 and 3.3 to 4.5, respectively. In addition, filtering the samples had the benefit of decreasing the variation between repeated measurements for the *p*LDH portion of the assay (*F* test, *P* = 0.02).

After filtration, the SCSD ELISA was performed on DBS extracts. The previously reported protocol was performed directly in lysed whole blood.^19^ Because DBS extracts are more dilute than lysed whole blood, blocking conditions for the assay had to be re-optimized. It was found that adding an equal volume of 10% nonfat dried milk to DBS extracts resulted in the highest signal-to-noise ratio (Supplemental Figure 2A). Magnetic bead volumes and detection antibody concentrations used in the SCSD ELISA for *p*LDH and HRP2 were screened in this new matrix, and it was found that the optimized conditions for these parameters were identical to those in the original protocol for both biomarkers (Supplemental Figure 2B and C).

### Dried blood spot SCSD ELISA performance.

**Figure 2.**
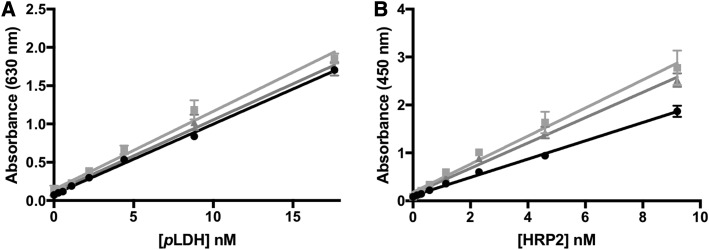
Standard curves for dried blood spot on-bead simultaneous capture and sequential detection ELISA for (**A**) *Plasmodium* lactate dehydrogenase (*p*LDH) and (**B**) histidine-rich protein 2 (HRP2).

The performance of the DBS SCSD ELISA protocol was evaluated using DBS made from parasitized whole blood. The assay was performed in triplicate once per day for 3 days (Figure 2). The linear range of the assay was found to be 0.6–18 nM for *p*LDH and 0.15–9.5 nM for HRP2. The intra-assay variation was 10.5% for *p*LDH and 4.7% for HRP2. The inter-assay variation was 12.5% for *p*LDH and 16.9% for HRP2. All four %CV values demonstrate acceptable reproducibility. The limits of detection were 600 ± 500 pM *p*LDH and 69 ± 30 pM HRP2, corresponding to 150 and 24 parasites/μL in our in-house culture, respectively. It is important to note that these lower limits are reported as the biomarker concentrations in the original whole blood sample that was spotted onto the DBS card. Thus, the inherent dilution associated with DBS extraction and DBS extraction efficiency are taken into account. Although intended for laboratory use, the performance of the DBS SCSD ELISA was equal to or better than that of currently available malaria rapid diagnostic tests.

### Biomarker detectability over time.

Dried blood spot cards are designed for long-term storage and preservation of biological samples. However, it has been shown that biomarker detectability from DBS changes over time.^16,25^ Thus, we measured *p*LDH and HRP2 signals from negative and positive (0 and 1,000 parasites/μL) DBS stored at both −20°C and room temperature over time. As shown in Supplemental Figure 3, neither *p*LDH nor HRP2 signal significantly changed after 6 months of storage at –20°C. However, for both biomarkers, recovery dramatically dropped over time when stored at room temperature. The *p*LDH signal at day 8 was reduced to 35% of the signal on day 0 and HRP2 signal was reduced to 31% in the same time period. This signal loss could be due to protein breakdown and loss of structure over time or to reduced extraction efficiency off the DBS card.

### Patient DBS samples from rural Zambia.

Dried blood spot samples were collected over 15 time points after treatment of 15 patients; in total, 225 DBSs were analyzed for this study. Parasitemias at each time point were determined by microscopy at the time of collection. Because DBS patient samples were 1–2 years old when analyzed, it was not assumed that the extraction efficiency of these patient samples would be the same as that of freshly prepared DBS standards. Thus, rather than comparing patient DBS to a standard curve of freshly prepared DBS to determine biomarker concentrations in the original whole blood sample that was spotted onto the card, biomarker concentrations in extracts were determined. This was performed at Macha Research Trust using standard curves in parasitized whole blood diluted 1:19, approximating the DBS extract matrix. Several assays were performed each day over the course of 2 weeks (*N* = 14). The intra-assay variation was 9.2% for *p*LDH and 6.1% for HRP2 and the inter-assay variation was 19.2% and 24.5% for *p*LDH and HRP2, respectively. Linear ranges for the assay were 10–520 pM *p*LDH and 10–680 pM HRP2. The limit of detection for the *p*LDH portion of the assay was 9 ± 6 pM, corresponding to six parasites/μL in our in-house culture. The detection limit for HRP2 was 7 ± 6 pM, which corresponds to four parasites/μL. These detection limits were used as cutoff values for determination of positive patient samples.

**Figure 3.**
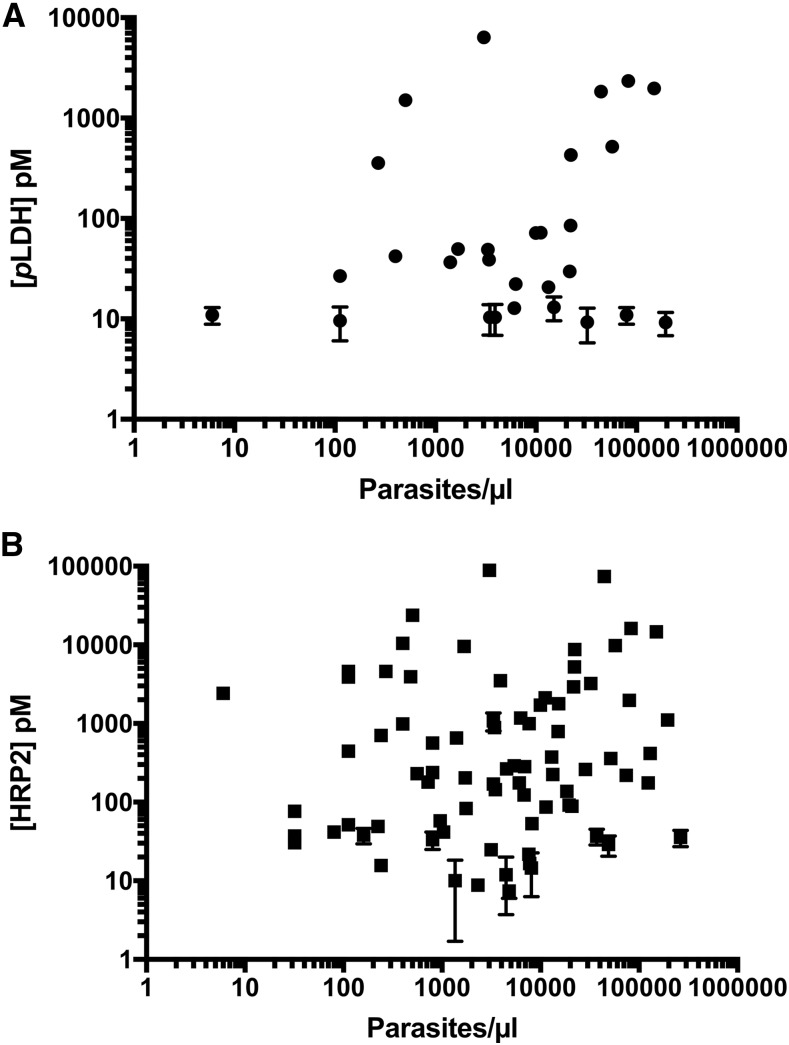
Correlations between (**A**) *Plasmodium* lactate dehydrogenase (*p*LDH) and parasitemia and (**B**) histidine-rich protein 2 (HRP2) and parasitemia in patient samples from rural Zambia. Weak correlations between biomarker concentrations and parasite burdens were observed. Note: for many data points, error bars are smaller than the size of the symbol representing the mean value.

The relationships between biomarker concentrations and parasite levels for both *p*LDH and HRP2 based on all DBS patient samples analyzed in this study are shown in Figure 3. Similar to previous reports, HRP2 concentrations were several orders of magnitude higher than *p*LDH concentrations.^26^ Weak-to-moderate, but significant (*P* < 0.001), positive correlations with parasitemia were observed for both biomarkers. The Spearman correlation coefficient was 0.36 (0.23–0.47) for *p*LDH and parasitemia and 0.46 (0.36–0.57) for HRP2 and parasitemia, demonstrating the utility of these biomarkers for malaria diagnosis. The nonparametric Spearman correlation coefficient was chosen because the parasite densities and concentrations measured do not follow normal distributions (D’Agostino and Pearson normality tests, *P* < 0.001). For *p*LDH, direct correlations with parasitemia have been demonstrated in the literature for both *P. falciparum* and *Plasmodium vivax* malaria.^26–28^ The strength of the correlation found in this study is lower than that in some reports, possibly because of the lack of controlled DBS storage conditions after sample collection. Previous reports have shown that uncontrolled DBS storage can lead to reduced *p*LDH recovery in mock patient samples.^25^ Similarly, we found that DBS storage at room temperature resulted in a drastic reduction of *p*LDH detectability, potentially explaining why DBS from seven patients with high initial parasitemia had initial *p*LDH levels near or below the detection limit of the DBS SCSD ELISA. However, biological factors, such as parasite life cycle stage, also affect *p*LDH expression, potentially influencing the strength of the observed correlation.^29^

Many studies have shown correlation between HRP2 and parasitemia, although some have found no correlation.^16,26,30^ While uncontrolled storage conditions have a similar detrimental impact on HRP2 detectability as for *p*LDH, initial HRP2 concentrations were detectable for all patients in this study. However, HRP2 expression has been shown to vary with parasite stage and strain.^31,32^ In addition, the duration of infection and persistence of HRP2 in circulation, addressed in detail in the next section, likely influenced the strength of the correlation between biomarker concentration and parasite density.

### Biomarker clearance.

**Figure 4.**
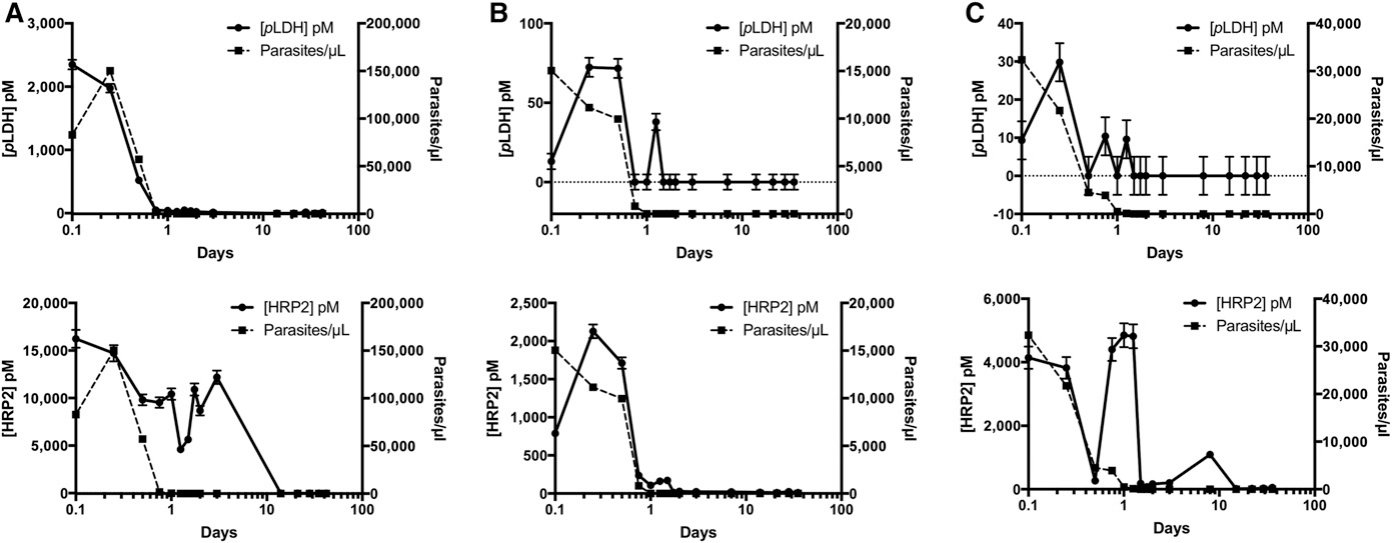
Biomarker clearance trends for three representative patients collected over 35 days. *Plasmodium* lactate dehydrogenase (*p*LDH) (top, solid) and histidine-rich protein 2 (HRP2) (bottom, solid) in dried blood spot extract and parasitemia (dashed) are plotted against time for (**A**) Patient 29, (**B**) Patient 55, and (**C**) Patient 58.

Unique parasite and biomarker clearance patterns were observed for each patient. Figure 4 shows clearance rates for three representative patients and Supplemental Figure 4 shows clearance rates for the remaining 12 patients. Overall, for the 15 patients in this study, the median parasite clearance time by microscopy after treatment with artemether–lumefantrine was 30 hours (interquartile range: 24–36 hours). Clearance times for the biomarkers were determined as the first time point in which the biomarker was undetectable for that time point and all subsequent time point measurements. The median *p*LDH clearance time was 36 hours (interquartile range: 6–72 hours) after treatment, following closely with parasite clearance time. In contrast, 13 of the 15 patients (87%) had measurable HRP2 levels at the final time point of this study (35–52 days after treatment). It should be noted that five of the 13 patients (38%) who were HRP2 positive at the last time point had undetectable HRP2 levels at least once at a previous time point. It is possible that uncontrolled storage conditions may have contributed to the undetectable HRP2 levels in the earlier time points. However, for one patient, *p*LDH levels also increased at the final time point, indicating possible reinfection or recrudescence (Supplemental Table 1, Patient 30).

The relationship between intensity of infection and biomarker persistence was also investigated. Patient infection levels were classified based on initial parasitemias: low (0–14,999 parasites/μL, *N* = 7), medium (15,000–74,999 parasites/μL, *N* = 5), and high (≥ 75,000 parasites/μL, *N* = 3). Using a one-way ANOVA (df = 12), there were no significant differences in parasite clearance time (*P* = 0.4221), *p*LDH clearance time (*P* = 0.5543), or HRP2 clearance time (*P* = 0.3206) across all three groups.

**Figure 5.**
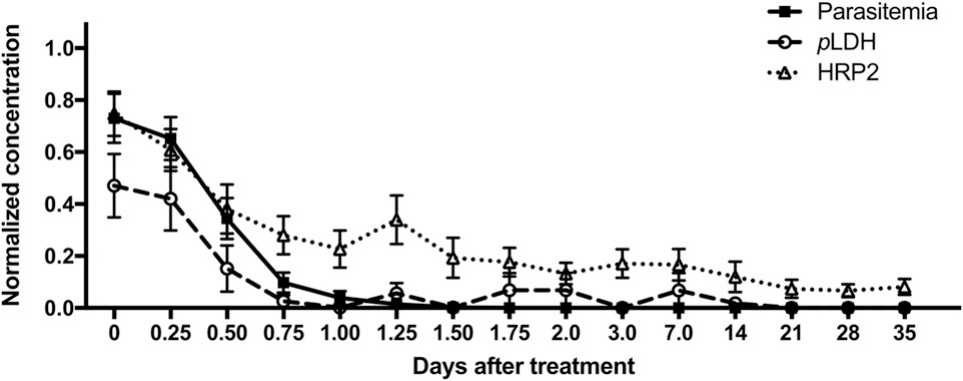
Normalized parasite, *Plasmodium* lactate dehydrogenase (*p*LDH), and histidine-rich protein 2 (HRP2) clearance patterns plotted over time.

The overall clearance patterns for all patients are represented in Figure 5. For each patient, parasite and biomarker levels were normalized to the highest concentration measured. The average across all patients at each time point was calculated and plotted, allowing for a clear visualization of the overall parasite, *p*LDH, and HRP2 clearance patterns in this study. In general, *p*LDH became undetectable before infections became submicroscopic. In contrast, HRP2 remained in circulation for the duration of the study, decreasing in concentration slowly over time. The persistence and accumulation of HRP2 in circulation over the duration of an infection likely explain why HRP2 concentrations were significantly higher than *p*LDH concentrations for all patients in this study.

In the context of malaria elimination, an ideal evaluation tool would be positive when a patient has an active infection and negative in the absence of parasites. Distinguishing between *falciparum* and non-*falciparum* infections would also be clinically useful and inform treatment and provide useful epidemiological data. A sensitive dual *p*LDH and HRP2 detection method could fulfill these ideals; however, this work highlights the challenges of developing a dual assay. Although *p*LDH detection could overcome the lack of specificity of HRP2 that results from persistence in host circulation after parasite clearance, the relatively low levels of circulating *p*LDH mean that active infections could be missed in the dual format. Such a result would undermine malaria elimination efforts, allowing active infections to persist and contribute to transmission. In contrast, an HRP2-only diagnostic could result in overdiagnosis and treatment, potentially resulting in unnecessary costs and a failure to treat other serious illnesses. Thus, there is a pressing need to develop more sensitive molecular recognition elements and detection methods for *p*LDH.

The previously developed SCSD ELISA fills this need and is an order of magnitude more sensitive than commercially available ELISA kits for both *p*LDH and HRP2.^19^ Here, the assay was adapted for detection of both biomarkers from a single DBS. Uncontrolled DBS storage conditions and the inherent dilution when DBSs are extracted into buffer contributed to reduced analytical sensitivity of the assay, although detection limits of the DBS SCSD ELISA remain comparable to that of commercially available ELISA kits for *p*LDH and HRP2 applied to whole blood samples. In contrast to commercially available ELISA kits, which are singleplex and take 3–6 hours to complete, the SCSD ELISA is capable of detecting two biomarkers from a single DBS in less than 1 hour. In addition, the ease with which the antibody-functionalized beads were transported to Zambia and the possibility of lyophilizing the beads for stable ambient storage could allow the developed assay to be applied in laboratories in low-resource settings. Thus, the DBS SCSD ELISA has the potential to increase the throughput and information yield of large epidemiological or surveillance studies based on DBS samples. To this end, we have demonstrated the utility of this assay for the evaluation of biomarker clearance in a patient population from rural Zambia. In the future, the DBS SCSD ELISA will be useful for the characterization of clearance patterns in other populations and could also serve as a rapid, preliminary screening tool for parasites with *pfhrp2* deletions.

## CONCLUSION

In this work, the on-bead SCSD ELISA for *p*LDH and HRP2 was adapted for use with DBS samples. For mock DBS samples, the assay was highly reproducible and could detect *p*LDH as low as 600 ± 500 pM and HRP2 as low as 69 ± 30 pM, corresponding to 150 and 24 parasites/μL in our in-house culture, respectively. Using the DBS SCSD ELISA, we demonstrated the need for controlled DBS storage; the detectability of both *p*LDH and HRP2 from DBS decreased nearly 70% after 8 days of storage at room temperature. Next, we applied the DBS SCSD ELISA to patient DBS samples from rural Zambia to measure *p*LDH and HRP2. In these samples, weak-to-moderate correlations between biomarker concentration and parasite density were found for both biomarkers, and the overall concentrations of HRP2 were several orders of magnitude higher than those of *p*LDH. Finally, biomarker clearance patterns relative to parasite clearance were studied. It was found that *p*LDH clearance followed closely with parasite clearance, whereas 87% of patients had detectable levels of HRP2 for 35–52 days after treatment. This work demonstrated the utility of the SCSD ELISA for quantifying *p*LDH and HRP2 from DBS samples and its potential for future application in epidemiological studies.

## Supplementary Material

Supplemental text, figures and table
