# Supplementary material for: Characterization of Plasmodium Lactate Dehydrogenase and Histidine-Rich Protein 2 Clearance Patterns via Rapid On-Bead Detection from a Single Dried Blood Spot
Source: Am J Trop Med Hyg. 2018 Mar 19;98(5):1389–96. doi: 10.4269/ajtmh.17-0996 (PMC5953395; doi:10.4269/ajtmh.17-0996)
Supplement: Supplementary file 1 [file tpmd170996.SD1.pdf]

## Supplemental text 1

**Plate ELISA for *Plasmodium* lactate dehydrogenase standardization in in-house D6 culture.** First, 100 µL of 2 µg/mL 19g7 in PBS was placed in a 96-well plate and incubated for 1 hour. The plate was washed three times with 250 µL of 1× PBS with 0.1% Tween-20 (PBST). Next, 250 µL of 15% nonfat dried milk in PBST was added to each well and the plate was incubated for 2 hours. After washing three times with PBST, 100 µL of standard rc*Pf*LDH and in-house culture diluted in PBST with 0.1% nonfat dried milk were placed on the plate and incubated for 2 hours. The plate was then washed five times with PBST, and 100 µL of 2 µg/mL 1201: HRPx in PBST with 0.5% nonfat dried milk was added to each well and the plate was incubated for 1 hour. After washing five times with PBST, 100 µL of TMB One solution was added to the wells, the plate was incubated for 15 minutes, and the reaction was quenched with 2M $H_2SO_4$. Absorbance was measured at 450 nm.

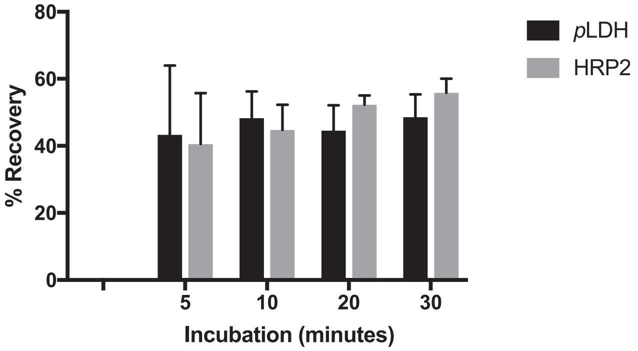


Supplemental Figure 1. Recovery of *Plasmodium* lactate dehydrogenase (*p*LDH) and histidine-rich protein 2 (HRP2) as a function of dried blood spot incubation time in PBS with 0.1% Tween-20. No significant differences were found between extraction efficiencies of *p*LDH and HRP2, and 10 minutes was chosen as the optimum incubation time.

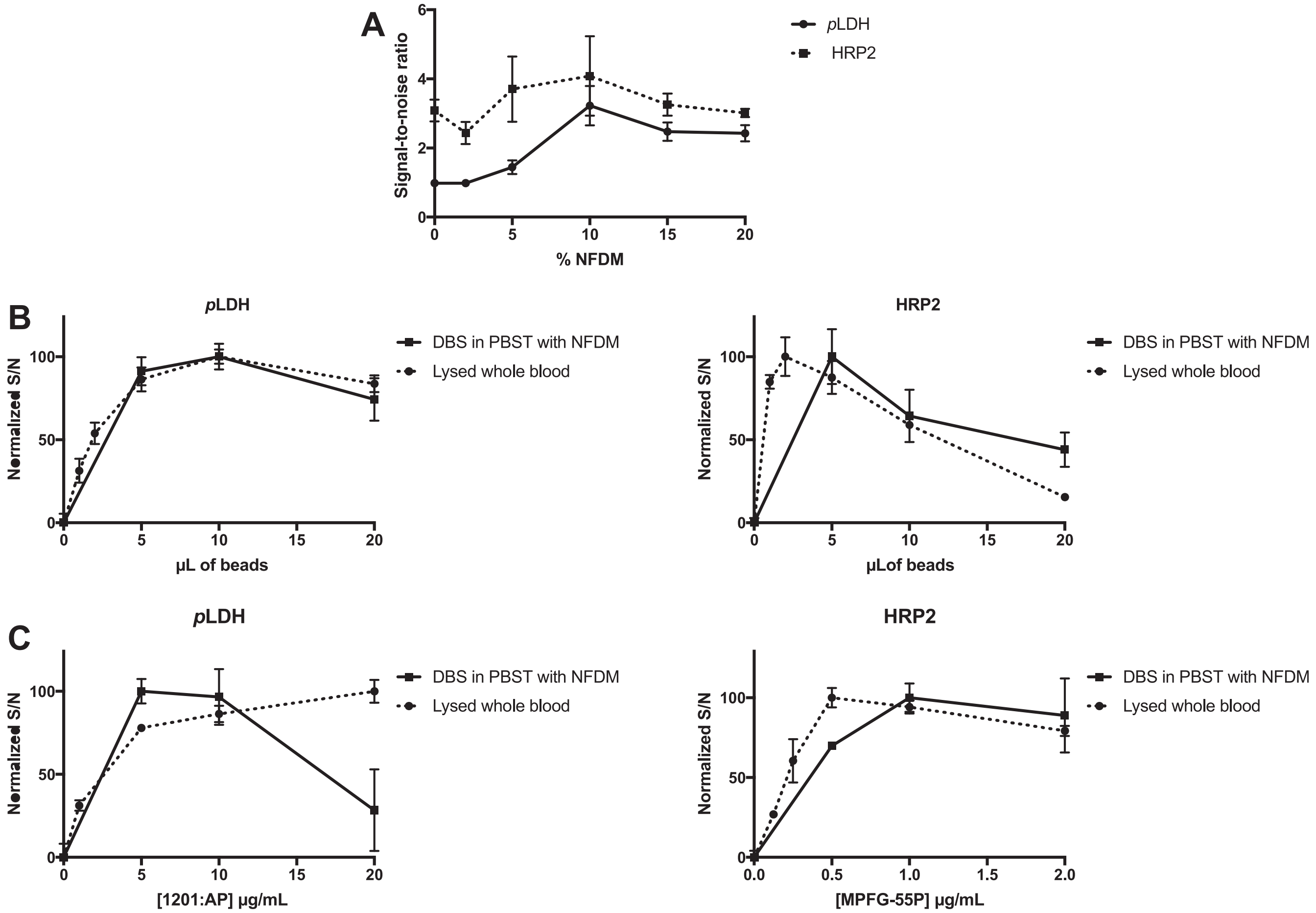


SUPPLEMENTAL FIGURE 2. Optimization of (**A**) nonfat dried milk concentration in blocking solution, (**B**) antibody-functionalized magnetic bead volume for *Plasmodium* lactate dehydrogenase (*p*LDH) (left) and histidine-rich protein 2 (HRP2) (right), and (**C**) detection antibody concentration for LDH (left) and HRP2 (right).

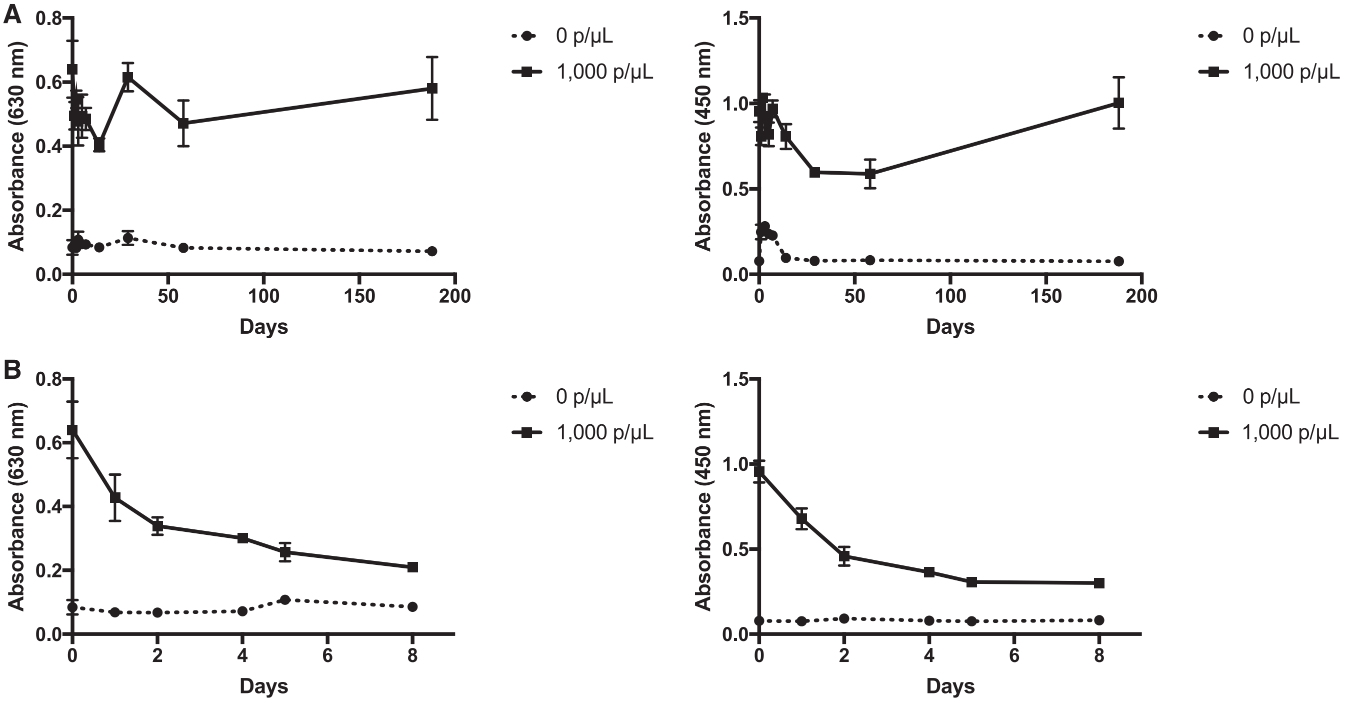


Supplemental Figure 3. Dried blood spot (DBS) storage time at (**A**) −20°C and (**B**) room temperature vs. DBS simultaneous capture and sequential detection ELISA signal for *Plasmodium* lactate dehydrogenase (left) and histidine-rich protein 2 (right).

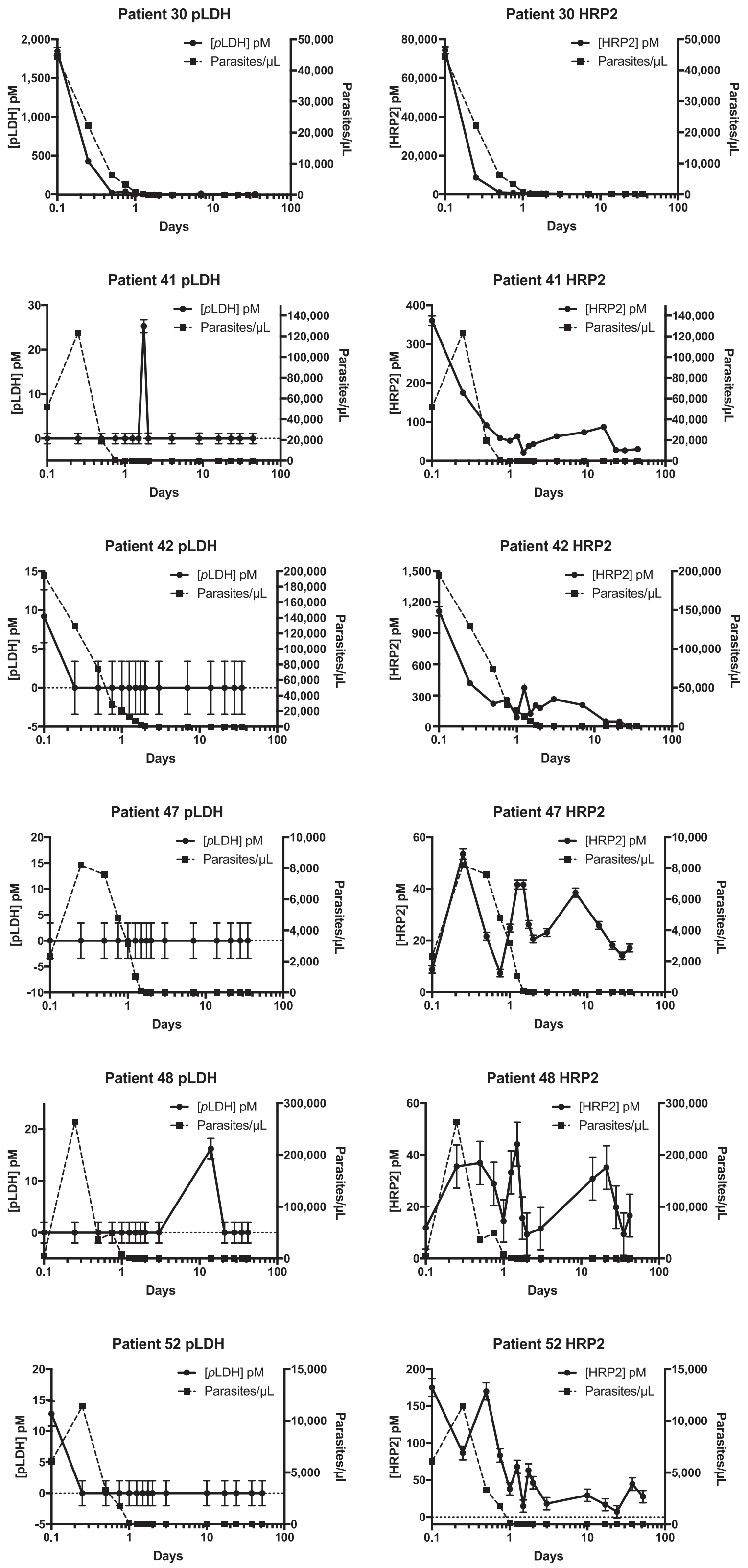


Supplemental Figure 4. Biomarker clearance patterns for individual patients from rural Zambia.

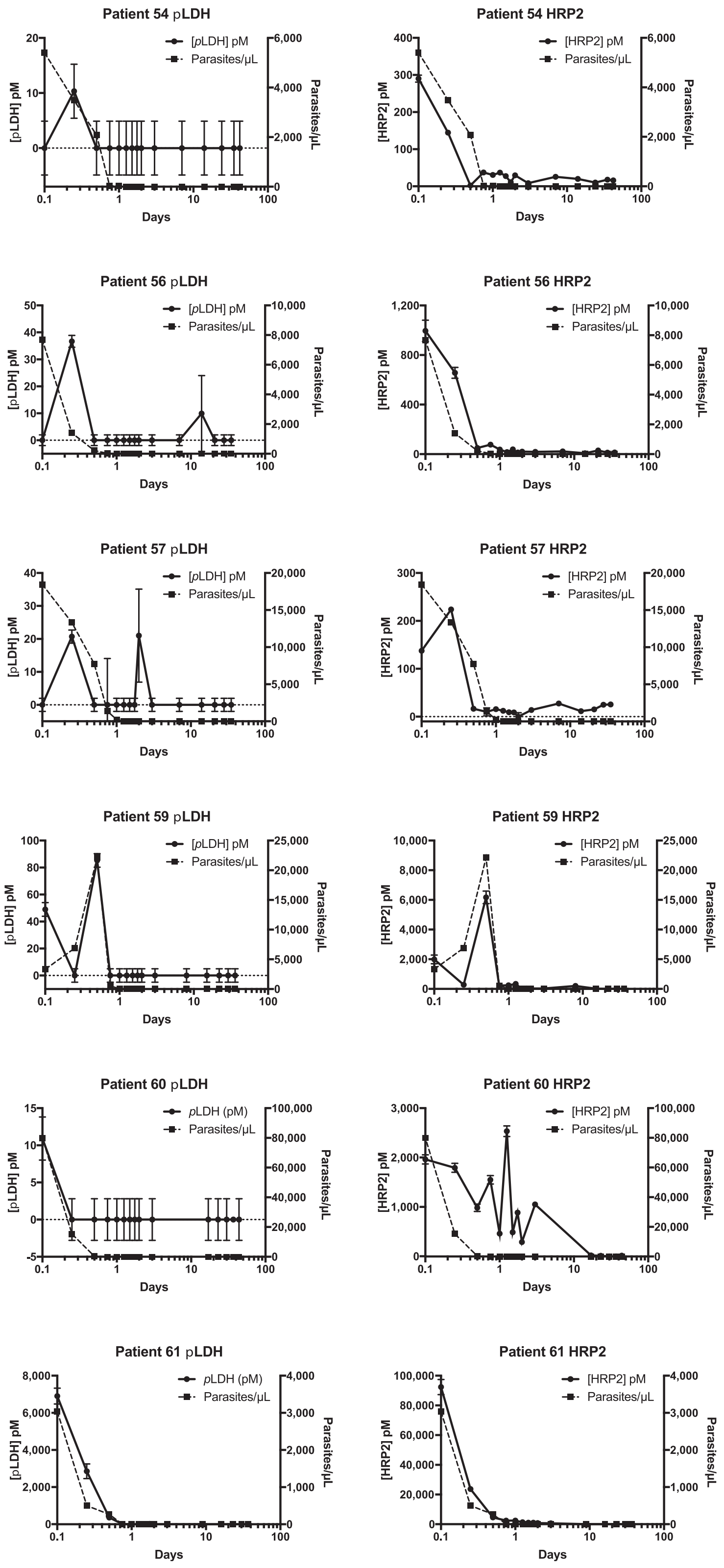


Supplemental Figure 4. (Continued)

Supplemental Table 1

Patient biomarker concentrations over time

| Patient ID | Time (days) | Parasites/μL | *p*LDH (pM) | | HRP2 (pM) | |
|---|---|---|---|---|---|---|
| | | | Average | Error | Average | Error |
| Patient 29 | 0 | 82,880 | 2,350 | 70 | 16,200 | 900 |
| | 0.25 | 150,189 | 1,980 | 70 | 14,700 | 900 |
| | 0.5 | 57,258 | 520 | 13 | 9,800 | 600 |
| | 0.75 | 1,680 | 49.6 | 2.0 | 9,500 | 600 |
| | 1 | 400 | 42.0 | 1.9 | 10,400 | 600 |
| | 1.25 | 112 | 26.8 | 1.7 | 4,600 | 300 |
| | 1.5 | 0 | 50.0 | 2.0 | 5,600 | 300 |
| | 1.75 | 0 | 35.9 | 1.8 | 10,900 | 600 |
| | 2 | 0 | 22.8 | 1.6 | 8,688.4 | 500 |
| | 3 | 0 | 13 | 1.6 | 12,200 | 709.4 |
| | 14 | 0 | 0 | 1.5 | 0 | 8 |
| | 21 | 0 | 0 | 1.5 | 0 | 8 |
| | 28 | 0 | 16.3 | 1.6 | 0 | 8 |
| | 35 | 0 | 0 | 1.5 | 0 | 8 |
| | 42 | 0 | 11.6 | 1.6 | 0 | 8 |
| Patient 30 | 0 | 44,400 | 1,840 | 50 | 74,000 | 2,000 |
| | 0.25 | 22,194 | 428 | 14 | 8,800 | 200 |
| | 0.5 | 6,280 | 22.3 | 1.4 | 1,180 | 40 |
| | 0.75 | 3,400 | 38.8 | 1.8 | 890 | 30 |
| | 1 | 800 | 0 | 1.2 | 564 | 19 |
| | 1.25 | 240 | 0 | 1.2 | 700 | 20 |
| | 1.5 | 112 | 0 | 1.2 | 446 | 15 |
| | 1.75 | 0 | 0 | 1.2 | 562 | 19 |
| | 2 | 0 | 0 | 1.2 | 534 | 18 |
| | 3 | 0 | 0 | 1.2 | 442 | 15 |
| | 7 | 0 | 14.7 | 1.3 | 134 | 5 |
| | 14 | 0 | 0 | 1.2 | 14.9 | 2.0 |
| | 21 | 0 | 0 | 1.2 | 0 | 2.3 |
| | 28 | 0 | 0 | 1.2 | 0 | 2.3 |
| | 35 | 0 | 10.1 | 1.2 | 22.9 | 2.4 |
| Patient 41 | 0 | 51,480 | 0 | 1.2 | 360 | 12 |
| | 0.25 | 123,579 | 0 | 1.2 | 175 | 6 |
| | 0.5 | 19,440 | 0 | 1.2 | 92 | 4 |
| | 0.75 | 960 | 0 | 1.2 | 58 | 3 |
| | 1 | 112 | 0 | 1.2 | 52 | 3 |
| | 1.25 | 0 | 0 | 1.2 | 63 | 3 |
| | 1.5 | 0 | 0 | 1.2 | 21.6 | 2.4 |
| | 1.75 | 0 | 25.3 | 1.4 | 38 | 3 |
| | 2 | 0 | 0 | 1.2 | 43 | 3 |
| | 4 | 0 | 0 | 1.2 | 63 | 3 |
| | 9 | 0 | 0 | 1.2 | 73 | 3 |
| | 16 | 0 | 0 | 1.2 | 87 | 4 |
| | 23 | 0 | 0 | 1.2 | 27 | 3 |
| | 30 | 0 | 0 | 1.2 | 26 | 3 |
| | 44 | 0 | 0 | 1.2 | 30 | 3 |
| Patient 42 | 0 | 194,840 | 9 | 3 | 1,110 | 40 |
| | 0.25 | 129,298 | 0 | 3 | 417 | 11 |
| | 0.5 | 74,267 | 0 | 3 | 220 | 6 |
| | 0.75 | 28,480 | 0 | 3 | 260 | 7 |
| | 1 | 20,840 | 0 | 3 | 89 | 3 |
| | 1.25 | 12,960 | 0 | 3 | 373 | 10 |
| | 1.5 | 6,800 | 0 | 3 | 123 | 4 |
| | 1.75 | 1,720 | 0 | 3 | 204 | 6 |
| | 2 | 720 | 0 | 3 | 180 | 5 |
| | 3 | 0 | 0 | 3 | 264 | 7 |
| | 7 | 0 | 0 | 3 | 209 | 6 |
| | 14 | 0 | 0 | 3 | 49.2 | 1.9 |
| | 21 | 0 | 0 | 3 | 47.8 | 1.9 |
| | 28 | 0 | 0 | 3 | 0 | 1.4 |
| | 35 | 0 | 0 | 3 | 7.7 | 1.4 |
| Patient 47 | 0 | 2,320 | 0 | 3 | 8.8 | 1.4 |
| | 0.25 | 8,200 | 0 | 3 | 53.4 | 2.0 |
| | 0.5 | 7,600 | 0 | 3 | 21.7 | 1.5 |
| | 0.75 | 4,800 | 0 | 3 | 7.4 | 1.4 |
| | 1 | 3,160 | 0 | 3 | 24.8 | 1.5 |
| | 1.25 | 1,040 | 0 | 3 | 41.6 | 1.8 |
| | 1.5 | 80 | 0 | 3 | 41.6 | 1.8 |
| | 1.75 | 0 | 0 | 3 | 26.2 | 1.5 |

*(continued)*

Supplemental Table 1
Continued

| Patient ID | Time (days) | Parasites/μL | *p*LDH (pM) | | HRP2 (pM) | |
|---|---|---|---|---|---|---|
| | | | Average | Error | Average | Error |
| | 2 | 0 | 0 | 3 | 20.6 | 1.5 |
| | 3 | 0 | 0 | 3 | 23.1 | 1.5 |
| | 7 | 0 | 0 | 3 | 38.5 | 1.7 |
| | 14 | 0 | 0 | 3 | 25.9 | 1.5 |
| | 21 | 0 | 0 | 3 | 18.1 | 1.5 |
| | 28 | 0 | 0 | 3 | 14.1 | 1.4 |
| | 35 | 0 | 0 | 3 | 17.2 | 1.5 |
| Patient 48 | 0 | 4,480 | 0 | 2.0 | 12 | 8 |
| | 0.25 | 263,530 | 0 | 2.0 | 36 | 8 |
| | 0.5 | 37,212 | 0 | 2.0 | 37 | 8 |
| | 0.75 | 48,989 | 0 | 2.0 | 29 | 8 |
| | 1 | 8,080 | 0 | 2.0 | 15 | 8 |
| | 1.25 | 800 | 0 | 2.0 | 33 | 8 |
| | 1.5 | 0 | 0 | 2.0 | 44 | 8 |
| | 1.75 | 0 | 0 | 2.0 | 16 | 8 |
| | 2 | 0 | 0 | 2.0 | 9 | 8 |
| | 3 | 0 | 0 | 2.0 | 12 | 8 |
| | 14 | 0 | 16.2 | 2.0 | 31 | 8 |
| | 21 | 0 | 0 | 2.0 | 35 | 8 |
| | 28 | 0 | 0 | 2.0 | 20 | 8 |
| | 35 | 0 | 0 | 2.0 | 9 | 8 |
| | 42 | 0 | 0 | 2.0 | 17 | 8 |
| Patient 52 | 0 | 6,080 | 12.8 | 2.0 | 175 | 12 |
| | 0.25 | 11,400 | 0 | 2.0 | 87 | 9 |
| | 0.5 | 3,320 | 0 | 2.0 | 170 | 13 |
| | 0.75 | 1,760 | 0 | 2.0 | 83 | 9 |
| | 1 | 160 | 0 | 2.0 | 38 | 8 |
| | 1.25 | 0 | 0 | 2.0 | 68 | 8 |
| | 1.5 | 0 | 0 | 2.0 | 15 | 8 |
| | 1.75 | 0 | 0 | 2.0 | 63 | 8 |
| | 2 | 0 | 0 | 2.0 | 46 | 8 |
| | 3 | 0 | 0 | 2.0 | 18 | 8 |
| | 10 | 0 | 0 | 2.0 | 29 | 8 |
| | 17 | 0 | 0 | 2.0 | 17 | 8 |
| | 24 | 0 | 0 | 2.0 | 7 | 8 |
| | 38 | 0 | 0 | 2.0 | 45 | 8 |
| | 52 | 0 | 0 | 2.0 | 28 | 8 |
| Patient 54 | 0 | 5,400 | 0 | 5 | 290 | 9 |
| | 0.25 | 3,480 | 10 | 5 | 145 | 5 |
| | 0.5 | 2,080 | 0 | 5 | 0 | 0.9 |
| | 0.75 | 32 | 0 | 5 | 37.3 | 1.5 |
| | 1 | 32 | 0 | 5 | 30.6 | 1.3 |
| | 1.25 | 0 | 0 | 5 | 36.9 | 1.5 |
| | 1.5 | 0 | 0 | 5 | 27.5 | 1.3 |
| | 1.75 | 0 | 0 | 5 | 10.8 | 1.0 |
| | 2 | 0 | 0 | 5 | 29.5 | 1.3 |
| | 3 | 0 | 0 | 5 | 8.4 | 0.9 |
| | 7 | 0 | 0 | 5 | 25.7 | 1.2 |
| | 14 | 0 | 0 | 5 | 19.7 | 1.1 |
| | 24 | 0 | 0 | 5 | 10.2 | 1.0 |
| | 35 | 0 | 0 | 5 | 17.9 | 1.1 |
| | 42 | 0 | 0 | 5 | 15.9 | 1.0 |
| Patient 55 | 0 | 15,040 | 13 | 5 | 790 | 40 |
| | 0.25 | 11,160 | 72 | 6 | 2,130 | 90 |
| | 0.5 | 9,960 | 72 | 6 | 1,710 | 80 |
| | 0.75 | 800 | 0 | 5 | 237 | 7 |
| | 1 | 0 | 0 | 5 | 107 | 4 |
| | 1.25 | 0 | 38 | 5 | 162 | 5 |
| | 1.5 | 0 | 0 | 5 | 171 | 5 |
| | 1.75 | 0 | 0 | 5 | 12.8 | 1.0 |
| | 2 | 0 | 0 | 5 | 28.2 | 1.3 |
| | 3 | 0 | 0 | 5 | 24.0 | 1.2 |
| | 7 | 0 | 0 | 5 | 20.9 | 1.1 |
| | 14 | 0 | 0 | 5 | 15.7 | 1.0 |
| | 21 | 0 | 0 | 5 | 11.9 | 1.0 |
| | 28 | 0 | 0 | 5 | 23.7 | 1.2 |
| | 35 | 0 | 0 | 5 | 11.0 | 1.0 |
| Patient 56 | 0 | 7,680 | 0 | 2.0 | 1,000 | 90 |

*(continued)*

Supplemental Table 1
Continued

| Patient ID | Time (days) | Parasites/μL | *p*LDH (pM) | | HRP2 (pM) | |
|---|---|---|---|---|---|---|
| | | | Average | Error | Average | Error |
| | 0.25 | 1,400 | 36.7 | 2.2 | 660 | 40 |
| | 0.5 | 224 | 0 | 2.0 | 49.0 | 1.3 |
| | 0.75 | 32 | 0 | 2.0 | 76.5 | 2.0 |
| | 1 | 0 | 0 | 2.0 | 37.9 | 1.0 |
| | 1.25 | 0 | 0 | 2.0 | 16.6 | 0.5 |
| | 1.5 | 0 | 0 | 2.0 | 38.1 | 1.0 |
| | 1.75 | 0 | 0 | 2.0 | 13.7 | 0.4 |
| | 2 | 0 | 0 | 2.0 | 20.6 | 0.6 |
| | 3 | 0 | 0 | 2.0 | 18.8 | 0.5 |
| | 7 | 0 | 0 | 2.0 | 22.6 | 0.6 |
| | 14 | 0 | 10 | 14 | 8 | 8 |
| | 21 | 0 | 0 | 2.0 | 29.5 | 0.8 |
| | 28 | 0 | 0 | 2.0 | 12.5 | 0.4 |
| | 35 | 0 | 0 | 2.0 | 13.5 | 0.4 |
| Patient 57 | 0 | 18,440 | 0 | 2.0 | 138 | 4 |
| | 0.25 | 13,320 | 20.7 | 2.0 | 224 | 6 |
| | 0.5 | 7,760 | 0 | 2.0 | 16.6 | 0.5 |
| | 0.75 | 1,360 | 0 | 14 | 10 | 8 |
| | 1 | 240 | 0 | 2.0 | 15.7 | 0.4 |
| | 1.25 | 0 | 0 | 2.0 | 12.3 | 0.4 |
| | 1.5 | 0 | 0 | 2.0 | 9.2 | 0.3 |
| | 1.75 | 0 | 0 | 2.0 | 9 | 0.3 |
| | 2 | 0 | 21 | 14 | 0 | 8 |
| | 3 | 0 | 0 | 2.0 | 13.9 | 0.4 |
| | 7 | 0 | 0 | 2.0 | 27.7 | 0.8 |
| | 14 | 0 | 0 | 2.0 | 11.7 | 0.3 |
| | 21 | 0 | 0 | 2.0 | 15.1 | 0.4 |
| | 28 | 0 | 0 | 2.0 | 24.9 | 0.7 |
| | 35 | 0 | 0 | 2.0 | 25.5 | 0.7 |
| Patient 58 | 0 | 32,360 | 9 | 5 | 3,200 | 300 |
| | 0.25 | 21,720 | 30 | 5 | 2,900 | 300 |
| | 0.5 | 4,520 | 0 | 5 | 266 | 4 |
| | 0.75 | 3,920 | 10 | 5 | 3,500 | 300 |
| | 1 | 480 | 0 | 5 | 3,900 | 300 |
| | 1.25 | 112 | 10 | 5 | 3,900 | 300 |
| | 1.5 | 0 | 0 | 5 | 182 | 3 |
| | 1.75 | 0 | 0 | 5 | 77 | 3 |
| | 2 | 0 | 0 | 5 | 166 | 3 |
| | 3 | 0 | 0 | 5 | 201 | 3 |
| | 8 | 0 | 0 | 5 | 910 | 70 |
| | 15 | 0 | 0 | 5 | 18 | 3 |
| | 22 | 0 | 0 | 5 | 23 | 2.0 |
| | 29 | 0 | 0 | 5 | 33 | 2.0 |
| | 36 | 0 | 0 | 5 | 54 | 2.0 |
| Patient 59 | 0 | 3,320 | 49 | 5 | 1,100 | 300 |
| | 0.25 | 6,880 | 0 | 5 | 283 | 4 |
| | 0.5 | 22,120 | 85 | 5 | 5,300 | 400 |
| | 0.75 | 560 | 0 | 5 | 230 | 4 |
| | 1 | 0 | 0 | 5 | 250 | 4 |
| | 1.25 | 0 | 0 | 5 | 339 | 4 |
| | 1.5 | 0 | 0 | 5 | 16 | 3 |
| | 1.75 | 0 | 0 | 5 | 25 | 3 |
| | 2 | 0 | 0 | 5 | 31 | 3 |
| | 3 | 0 | 0 | 5 | 27 | 3 |
| | 8 | 0 | 0 | 5 | 177 | 3 |
| | 15 | 0 | 0 | 5 | 22 | 3 |
| | 22 | 0 | 0 | 5 | 0 | 3 |
| | 29 | 0 | 0 | 5 | 14 | 3 |
| | 36 | 0 | 0 | 5 | 16 | 3 |
| Patient 60 | 0 | 79,840 | 11 | 3 | 1,960 | 90 |
| | 0.25 | 15,320 | 0 | 3 | 1,790 | 90 |
| | 0.5 | 400 | 0 | 3 | 980 | 80 |
| | 0.75 | 0 | 0 | 3 | 1,550 | 90 |
| | 1 | 0 | 0 | 3 | 460 | 40 |
| | 1.25 | 0 | 0 | 3 | 2,530 | 110 |
| | 1.5 | 0 | 0 | 3 | 490 | 40 |
| | 1.75 | 0 | 0 | 3 | 880 | 50 |
| | 2 | 0 | 0 | 3 | 290 | 40 |

*(continued)*

Supplemental Table 1
Continued

| Patient ID | Time (days) | Parasites/μL | *p*LDH (pM) | | HRP2 (pM) | |
|---|---|---|---|---|---|---|
| | | | Average | Error | Average | Error |
| | 3 | 0 | 0 | 3 | 1,050 | 50 |
| | 17 | 0 | 0 | 3 | 19 | 7 |
| | 23 | 0 | 0 | 3 | 12 | 7 |
| | 30 | 0 | 0 | 3 | 0 | 7 |
| | 37 | 0 | 0 | 3 | 0 | 7 |
| | 44 | 0 | 0 | 3 | 15 | 7 |
| Patient 61 | 0 | 3,035 | 6,400 | 300 | 89,000 | 5,000 |
| | 0.25 | 503 | 1,510 | 100 | 23,700 | 1,300 |
| | 0.5 | 269 | 360 | 60 | 4,600 | 200 |
| | 0.75 | 6 | 11 | 3 | 2,400 | 100 |
| | 1 | 0 | 0 | 3 | 2,420 | 90 |
| | 1.25 | 0 | 0 | 3 | 1,390 | 60 |
| | 1.5 | 0 | 0 | 3 | 1,010 | 50 |
| | 1.75 | 0 | 0 | 3 | 1,050 | 50 |
| | 2 | 0 | 0 | 3 | 750 | 40 |
| | 3 | 0 | 0 | 3 | 590 | 40 |
| | 9 | 0 | 0 | 3 | 18 | 7 |
| | 16 | 0 | 0 | 3 | 0 | 7 |
| | 23 | 0 | 0 | 3 | 0 | 7 |
| | 30 | 0 | 0 | 3 | 0 | 7 |
| | 37 | 0 | 0 | 3 | 0 | 7 |

HRP2 = histidine-rich protein 2; *p*LDH = *Plasmodium* lactate dehydrogenase.
